# Tuning the Consonance of Microscopic Neuro-Cardiac Interactions Allows the Heart Beats to Play Countless Genres

**DOI:** 10.3389/fphys.2022.841740

**Published:** 2022-02-22

**Authors:** Mauro Franzoso, Lolita Dokshokova, Libero Vitiello, Tania Zaglia, Marco Mongillo

**Affiliations:** ^1^Department of Biomedical Sciences, University of Padova, Padova, Italy; ^2^Department of Biology, University of Padova, Padova, Italy

**Keywords:** cardiomyocytes, sympathetic neurons, neuro-cardiac junction, signaling nanodomains, β-adrenoceptor, neuro-cardiac junction

## Abstract

Different from skeletal muscle, the heart autonomously generates rhythmic contraction independently from neuronal inputs. However, speed and strength of the heartbeats are continuously modulated by environmental, physical or emotional inputs, delivered by cardiac innervating sympathetic neurons, which tune cardiomyocyte (CM) function, through activation of β-adrenoceptors (β-ARs). Given the centrality of such mechanism in heart regulation, β-AR signaling has been subject of intense research, which has reconciled the molecular details of the transduction pathway and the fine architecture of cAMP signaling in subcellular nanodomains, with its final effects on CM function. The importance of mechanisms keeping the elements of β-AR/cAMP signaling in good order emerges in pathology, when the loss of proper organization of the transduction pathway leads to detuned β-AR/cAMP signaling, with detrimental consequences on CM function. Despite the compelling advancements in decoding cardiac β-AR/cAMP signaling, most discoveries on the subject were obtained in isolated cells, somehow neglecting that complexity may encompass the means in which receptors are activated in the intact heart. Here, we outline a set of data indicating that, in the context of the whole myocardium, the heart orchestra (CMs) is directed by a closely interacting and continuously attentive conductor, represented by SNs. After a roundup of literature on CM cAMP regulation, we focus on the unexpected complexity and roles of cardiac sympathetic innervation, and present the recently discovered Neuro-Cardiac Junction, as the election site of “SN-CM” interaction. We further discuss how neuro-cardiac communication is based on the combination of extra- and intra-cellular signaling micro/nano-domains, implicating neuronal neurotransmitter exocytosis, β-ARs and elements of cAMP homeostasis in CMs, and speculate on how their dysregulation may reflect on dysfunctional neurogenic control of the heart in pathology.

## Multiple Roles of Neuro-Muscular Innervation

It is well-established that cardiac and skeletal muscles are “striated muscles,” made for a large part of contractile cells [e.g., cardiomyocytes (CMs) and myofibers, respectively] which, although sharing the ability to contract, possess distinctive features. If we consider cell morphology, myofibers are tapered cells, with varied length depending on the muscle type (from about 7–40 mm), while CMs are smaller in size (average longitudinal and transversal diameters are about 150 and 30 mm, respectively), with a rod-shaped morphology (Adams and Schwartz, 1980). Additionally, while contraction is underlain, in both muscles, by the serial arrangement of sarcomeres along the cellular matrix, substrates of cell shortening, including sarcolemma, transverse-tubules (T-tubules), sarcoplasmic reticulum (SR), as well as mechanisms regulating contraction [mostly modulation of intracellular (Ca^2+^)], are muscle-type specific (Adams and Schwartz, 1980). Furthermore, it has been long known that, while skeletal muscles retain the ability to regenerate upon tissue damage, thanks to the presence of the satellite cells (Tedesco et al., 2010), the myocardium is a post-mitotic tissue and the irreversible exit of CMs from cell cycle makes the heart extremely vulnerable to damage (Lafontant and Field, 2006).

It is unquestioned that skeletal muscle contraction, finalized to voluntary movements, initiates upon the neurogenic input provided by the innervating motor neuron (MN), which releases acetylcholine at the Neuro-Muscular Junction (NMJ), a well-characterized neuromuscular synapse (Ruff, 2003). Differently, cardiac blood propulsion occurs independently from innervation, as the myocardium homes specialized cells, able to spontaneously generate repeated action potentials, which are organized into the conduction system and dictate the basal heart rhythm (His, 1949; DiFrancesco, 1995). Such difference is manifested by the continued rhythmic contractions of an explanted heart, when supplied with oxygen and nutrients, and well-exemplified by heart transplants which, although the organ is disconnected from central neurons, allow survival of many patients suffering from complex cardiovascular diseases (Scalco et al., 2021). In the physiologic heart, however, function is continuously modulated to meet the perfusional demands of the organism, both during daily activities and stresses, and such fundamental adaptation, encompassing both increased heart rate (i.e., chronotropic effect) and contractile force (i.e., inotropic effect), is mostly dependent on the activity of noradrenaline-releasing cardiac sympathetic neurons (Zaglia and Mongillo, 2017).

The roles of neurogenic components in skeletal and cardiac muscle physiology have somewhat settled with a simplified view, classifying MNs as the nervous activators of skeletal muscles, and autonomic neurons solely as heart modulators. Recent research, in both myology and cardiology, has, however, brought to light novel roles of sympathetic neurons (SNs) in both fields, blurring the divide between skeletal and cardiac muscle innervation. While SNs were identified several years ago, in anatomic studies, to account for a significant fraction of the neuronal types in nerves directed to hindlimb muscles (Straka et al., 2018; Di Bona et al., 2020), their initial role was restricted to the regulation of muscle blood vessel diameter, which is coherent with the adaptative response to physical exercise (Lombard and Cowley, 2012). Nonetheless, several recent reports, using refined imaging methods and pharmacologic approaches, have shown that SNs interact with muscle fibers and play initially unsuspected functions, ranging from the modulation of synaptic transmission between MNs and muscle cells, to the maintenance of morphological and functional stability of the NMJ (Khan et al., 2016; Straka et al., 2018, 2021). In addition, noradrenaline stimulation of muscle fibers leads to increase in contraction force, *via* PKA activation, and subsequent phosphorylation of downstream targets, involved in the mechanism of cell shortening (i.e., L-type Ca^2+^ channels; ryanodine receptors; Cairns and Dulhunty, 1993; Röder et al., 2009; Rudolf et al., 2013; Khan et al., 2016; Straka et al., 2021). Furthermore, noradrenaline release by muscle-innervating SNs regulates myocyte trophism, through activation of β2-adrenoceptors (β2-ARs), which modulate protein degradation, by negatively impinging on the expression of atrophic gene patterns (Navegantes et al., 2004; Rudolf et al., 2013). All of these effects, initially attributed to the trophic function of MNs, have brought light back to the sympathetic component of motor nerves, and established that skeletal muscle cells receive neurogenic input from two parallel and distinct systems, underlaying contraction (MNs), on the one hand, and functional and structural modulation (SNs), on the other.

Interestingly, parallel studies of neurogenic control of the heart have extended the role of sympathetic innervation beyond the confines of the stress response, coming into play only when more cardiac output is needed (i.e., fight-or-flight reaction, hemodynamic, or emotional stresses). Indeed, we and others have demonstrated that cardiac sympathetic innervation has additional constitutive functions, which include control of heart rate on a beat-to-beat basis (Zaza et al., 1991; Zaglia and Mongillo, 2017), and activation of signaling pathways regulating CM division, trophism and electrophysiological properties (Ogawa et al., 1992; Kanevskij et al., 2002; O’Connell et al., 2003; Zaglia et al., 2013; Kreipke and Birren, 2015; Pianca et al., 2019). As example, we identified that SN-dependent activation of CM β2-ARs is required to maintain the correct cardiac mass, by inhibiting protein degradation *via* the β2-AR/Akt/FOXO/ubiquitin ligase pathway, with a mechanism very similar to that active in skeletal muscles (Zaglia et al., 2013; Zaglia and Mongillo, 2017).

Such evidence supports that, while cardiac and skeletal muscles are unquestionably different for phenotypic and functional aspects, they are not so dissimilar with regards to the relationship with sympathetic nervous components. At this point, it is inevitable to think that a fundamental difference in the way skeletal and cardiac muscle interact with neurons is given by the structures underlaying such interactions. In fact, it has been known for a long time that MNs signal to the innervated fiber in a synaptic way, thanks to the NMJ (Acheson, 1948; Welsh, 1948; Del Castillo and Katz, 1956). The myocardium is instead considered orphan of synaptic contacts, and the common perception was that SNs activate CM β-ARs through noradrenaline which, once released, diffuses into the myocardial interstitium (Zaglia and Mongillo, 2017). In contrast with this model, our group recently demonstrated that also in the heart, neurogenic control of the myocardium is expressed thanks to the existence of structured intercellular interactions, called Neuro-Cardiac Junctions (NCJs), allowing SNs to communicate with target CMs in a synaptic fashion (Prando et al., 2018). Although the molecules building the NCJ are, at the time being, only scarcely identified, and may be different from those forming the NMJ (Shcherbakova et al., 2007; Prando et al., 2018), the final effect of both the NMJ and the NCJ is to ensure optimal efficiency in the neuro-muscular intercellular communication.

Starting from these base notes, this review will focus on the communication occurring between the single SN varicosity and the target CM, operating through intracellular and extracellular signaling microdomains, using the skeletal muscle and the NMJ as a benchmark. Furthermore, starting from the fine characterization of the NMJ structure, we will present a roundup of the literature identifying the molecular bricks which build the NCJ.

## Intracellular Compartmentation of β-Adrenoceptor Signaling in Cardiomyocytes

As stated above, although the process of excitation-contraction coupling occurs in CMs independently from neuronal inputs, heart function is continuously adapted to precisely match the organism requests. Such adaptation results from the integration, by the central nervous system, of signals from peripheral sensors of homeostatic (i.e., blood pressure, pH, osmolarity, and temperature), sensory (environmental) and emotional (intrinsic) inputs, and routed to the heart through afferences of the autonomic nervous system (Scalco et al., 2021). Neurogenic release of noradrenaline activates adrenoreceptors (ARs), which, in CMs, are mostly the β-subtypes, increasing intracellular (cAMP) *via* receptor-associated Adenylyl Cyclases. Ignition of the β-AR/cAMP pathway is, by no doubts, the mainspring of cardiac “fight-or-flight” reaction, ultimately causing positive chronotropic, lusitropic and inotropic effects, mostly through PKA-dependent phosphorylation of sarcomeric proteins and molecular players regulating Ca^2+^ dynamics (Li et al., 2000; Bers, 2002; Eisner et al., 2017). While such mechanisms have been described few decades ago, and acknowledged thereafter, the notion that β-ARs belong to the large family of G-protein coupled receptors (GPCR), many of which share the same type of G-protein and second messenger, has soon opened the question (broadly applicable to signaling biology in any cell type) of specificity of receptor responses. In other words, “how can CMs activate the proper response to a selective receptor stimulation (e.g., β1*-* vs. β2-AR), if signaling is transduced by the same second messenger (i.e., cAMP) shared with many other receptor types, eliciting different, and sometimes opposed, biological responses?”

**Figure 1:**
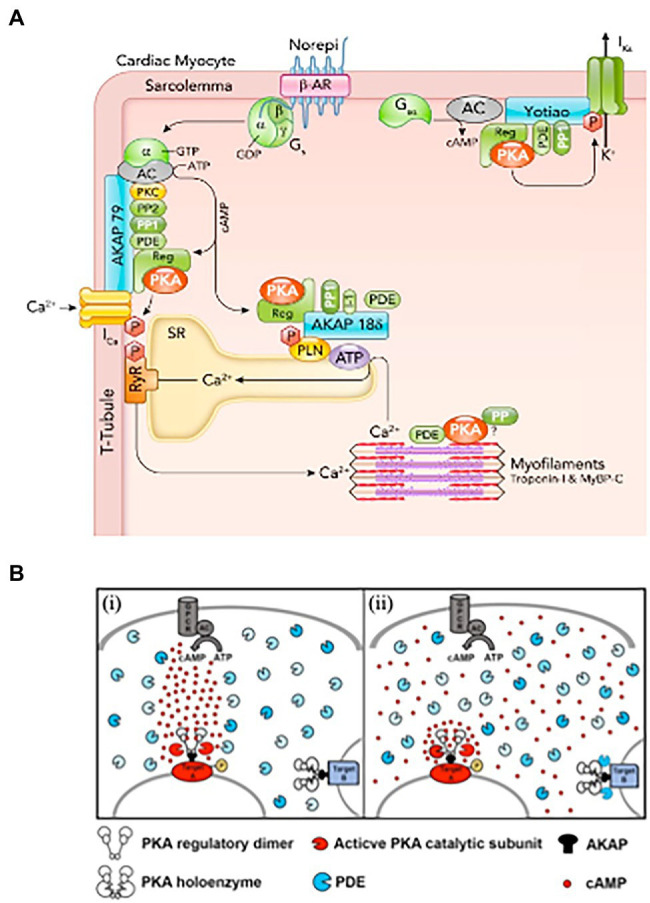
Activation of cardiomyocyte β-adrenoceptors lead to compartmentalized increase in (cAMP). **(A)** β-AR, cAMP, and PKA signaling proteins in cardiomyocytes. β-AR activated by norepinephrine (Norepi) causes adenylyl cyclase (AC) to produce cAMP, which binds the PKA regulatory subunit. Activated PKA can selectively phosphorylate nearby target proteins including sarcolemmal L-type Ca channels, which increases I_Ca_, SR phospholamban (PLN), which increases SR Ca-ATPase activity, and KCNQ1 to enhance delayed rectifier K^+^ current I_Ks_. PKA is anchored at these three targets by specific AKAPs (A-kinase anchoring proteins), namely AKAP150, AKAP18δ, and Yotiao. These AKAPs also bind additional cAMP-PKA modulators (PP1, PP2, PDEs, and PKC). **(B)** Hypotheses on the mechanisms underlying cAMP compartmentalization. (**i**) Restricted propagation model. On activation of the Gs protein-coupled receptor (GPCR), the adenylyl cyclase (AC) synthetizes cAMP. Phospodiesterases (PDEs), by degrading cAMP, limit its diffusion, directing propagation from the focal point of synthesis to a downstream PKA target (target A), leading to its selective phosphorylation. (**ii**) Nanodomain regulation model. cAMP freely propagates from the site of synthesis at the plasma membrane to the entire cell. PDEs anchored at specific subcellular sites act as a sink for cAMP, maintaining the level of the second messenger below the bulk cytosol and preventing activation of local PKA (exemplified by target B complex). At other sites, the absence of local PDEs and concomitant factors allow for cAMP to accumulate locally at levels higher than bulk cytosol, leading to selective phosphorylation (target A). According to this model, the functional outcome of the cAMP signal is dictated by the local levels of the second messenger in the extremely restricted environment immediately surrounding specific effector/target protein complexes. PDE, shown here in different shades of blue to illustrate the diversity of the multiple isoforms, localize to specific subcellular sites. **(A,B)** Modified with permission from Bers et al. (2019) and Chao et al. (2019).

In this context, seminal studies elegantly showed that activation of β-ARs in one side of the cell led to local enhancement of cAMP sensitive Ca^2+^ currents on the same cell portion, demonstrating the concept of compartmentalization of β-AR/cAMP signaling (Jurevičius and Fischmeister, 1996). The initial view of signal transduction as a linear chain of biochemical events, initiated from the plasma membrane and reaching all intracellular effector(s), was thus gradually shifted to a model whereby membrane receptors signal to a fraction of selected intracellular targets in their proximity, or partitioned in defined cellular regions (Steinberg and Brunton, 2001). Progress in biotechnology soon allowed deeper insight into these mechanisms, thanks to the development of biosensors enabling to monitor changes of (cAMP) in real-time, with high spatial resolution, in living cells. Use of early-generation, genetically encoded, cAMP biosensors showed that β-AR activation led to cAMP elevations in multiple discrete microdomains in cultured CMs, displaying in the living cell the concept of compartmentalized signaling (Zaccolo and Pozzan, 2002; Surdo et al., 2017). Subsequent studies demonstrated that local regulation of the second messenger within the cell results from the combination of different factors, including the cellular localization and activity of specific families of cAMP degrading enzymes, i.e., phosphodiesterases, physical barriers to cAMP diffusion and its buffering by PKA, altogether shaping the spatial distribution and kinetics of cAMP fluctuations, and its access to the intracellular targets (Zaccolo and Pozzan, 2002; Mongillo et al., 2004, 2006; Fischmeister et al., 2006; Saucerman et al., 2006; Vandecasteele et al., 2006; Soto et al., 2009). A relevant example of how spatial organization of cAMP responses reflects the stimulation of a specific receptor subtype comes from the study of Nikolaev et al. showing, in murine CMs, that β1- or β2-AR agonism elicits increases in cAMP which spread throughout the entire cytoplasm, or remain localized at the level of T-tubuli, respectively (Nikolaev et al., 2006). In incremental developments, the now-accepted concept of compartmentalization of cAMP has been delved into with increasing precision and detail, thanks to an extended toolkit of diverse cAMP and PKA biosensors, with enhanced sensitivity, or targeted to specific subcellular compartments, and the combination with strategies for local delivery of extracellular stimuli, subcellular uncaging of cAMP analogs or targeted Adenylyl Cyclases (Schleicher and Zaccolo, 2018; Ghigo and Mika, 2019). It is now well appreciated that specifically distributed Adenylyl Cyclases (that make cAMP) and specific phosphodiesterase isoforms (that degrade cAMP) are selectively localized in different cellular domains (Bers et al., 2019), including the plasma membrane, SR, nucleus and mitochondria (Mongillo and Zaccolo, 2006; Leroy et al., 2008; Mika et al., 2012; Stangherlin and Zaccolo, 2012; Bedioune et al., 2018; Ghigo and Mika, 2019). Such molecular arrangement is an uttermost factor allowing the same signaling molecule (cAMP) to control both the thundering effect of the “fight-or-flight” reaction and, independently, gene expression, mitochondrial dynamics or other subdued homeostatic processes (Mongillo et al., 2004, 2006; Saucerman et al., 2006; Nikolaev et al., 2010; Sample et al., 2012; Tsvetanova and von Zastrow, 2014; Li et al., 2015; Bers et al., 2019; Naim et al., 2019; Zaccolo et al., 2021; Figure 1).

Collective evidence from the studies on cAMP is that the second messenger dynamics and intracellular targets are regulated in a way far more complex than initially expected. Studies using targeted versions of cAMP sensors suggest that selective modulation of cAMP signaling may take place in confined sub-microscopic domains. The tight spatial regulation of cAMP gradients, and the specific localization of intracellular targets (e.g., PKA, Epac) and signal regulators (e.g., phosphodiesterases, protein phosphatases) have been shown to impact on the downstream effect of receptor activation (Zakhary et al., 2000; Rehmann et al., 2006). Adding to such complexity, recent advances, based on the integration of *in vitro* experiments and numerical modeling, show that, in addition to the amplitude and localization of the cAMP signal, its kinetics may result in unexpected effects, not entirely correlated to the second messenger concentration (Bers et al., 2019). In detail, activation of PKA, at precise subcellular compartments, reflects the potency and duration of β-AR stimulation with a non-linear dose/effect relationship, so that at lower level of β-AR activation, inotropy is enhanced, while recruitment of additional lusitropic benefit only occurs at higher β-AR activation levels (Bers et al., 2019). Of note, subsynaptic and sarcomeric cAMP/PKA microdomains have been described also in the skeletal muscle, where noradrenaline mainly activates β2-ARs with a predominant effect on gene activation (Röder et al., 2009; Rudolf et al., 2013).

**Figure 2:**
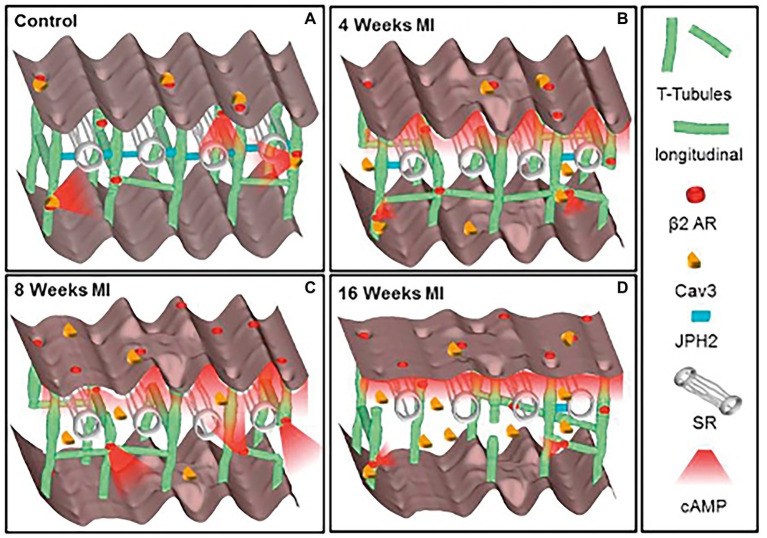
Schematic of the changes in structure and location of β2-ARs during heart failure progression. **(A)** In control CMs the *external* surface structure (Z-grooves and crests) and the *internal* transverse and axial tubules (TAT) network, are intact, and β2-AR are located exclusively on T-tubules, connected with the SR through Junctophilin 2 (JPH2). cAMP does not diffuse far from the site of β2-AR activation, and Caveolin-3 (Cav3) is predominantly on the cell membrane. In heart failure the surface structure deteriorates progressively. **(B)** Four weeks after myocardial infarction, JPH2 is downregulated, and the density of longitudinal elements increases. β2-AR dependent cAMP responses appear at the crest, and cAMP is no longer confined to the site of β2-AR activation. **(C,D)** At later time, the CM surface elements and T-tubule structure further deteriorate, with decreased density of longitudinal element. Notably, inefficient β2-AR dependent AC activation leads to significant reduction in cAMP production. Adapted with permission from Schobesberger et al. (2017).

Given the complex and meticulous regulation of the β-AR signaling pathway, which holds a fundamental role in heart physiology, it is not surprising that disruption of the fine space–time organization of the cAMP signal impacts on the adequate CM response to β-AR stimulation. Consistently, results from several studies, in both human and rodents, demonstrate that CMs from failing hearts feature dysregulation in β-AR/cAMP signaling, characterized by desensitization/downregulation of β-ARs, reduced cAMP synthesis and altered phosphorylation of the main PKA targets (Lohse et al., 2003; Zaccolo and Movsesian, 2007; Ghigo and Mika, 2019). In addition, by using Scanning Ion Conductance Microscopy, Gorelik and colleagues identified that submicroscopic alterations of cAMP regulation take place in failing heart cells, as result of structural CM changes, i.e., loss of membrane T-tubules (Nikolaev et al., 2010). Data from this and other research groups consistently demonstrated that in failing CMs, βs2-ARs are “uncoupled” from the localized pools of PKA,leroy which are physiologically targeted within the β2-AR/cAMP signaling compartment and, consequently, activation of β2-AR leads, in failing cells, to a wide cAMP propagation pattern similar to that observed in normal CMs upon β1-AR stimulation (Calaghan et al., 2008; Mehel et al., 2013; Schobesberger et al., 2017; Figure 2). Given this evidence, molecular tools tailored to restore the normal cAMP nanodomains are expectedly promising therapeutic strategies, applicable in several cardiac pathologies associated to dysfunctional neurogenic control of heart function (Leroy and Fischmeister, 2018).

Altogether, the examples described above support that the spatial and temporal regulation of signaling of one specific GPCR is restricted to tightly organized compartments within the cell and may occur with singular kinetics. This may represent the receptor “signature,” allowing its stimulation to distinctively activate the proper and specific biological responses in the target cell.

## The Reappraisal of the Idea of a “Cardiac Sympathetic Synapse”

While most studies on cAMP signaling, including those related to the fundamental mechanism underlying cardiac cell response to AR stimulation, have been conducted in cultured isolated cells, exposed to natural or artificial AR agonist, very few have aimed to replicate the conditions in which CMs are activated by SNs in the heart. Given that the complexity of cAMP responses, upon activation of β-ARs, depends on the cellular arrangement of the molecular partners of the signaling cascade, it is predictable that localization of the receptors throughout the heterogeneous cell membrane domains, the fraction—or specific subset—of receptors activated by the agonist, and the kinetics and potency of activation, are all critical players. As consequence, since SNs are the endogenous sources of noradrenaline triggering β-ARs in heart, to infer the implications of intracellular β-AR signaling compartmentation in neurocardiac physiology, the microanatomy of interactions between neurotransmitter-releasing neuronal varicosities and target cells, in the intact heart, must be taken into account.

**Figure 3:**
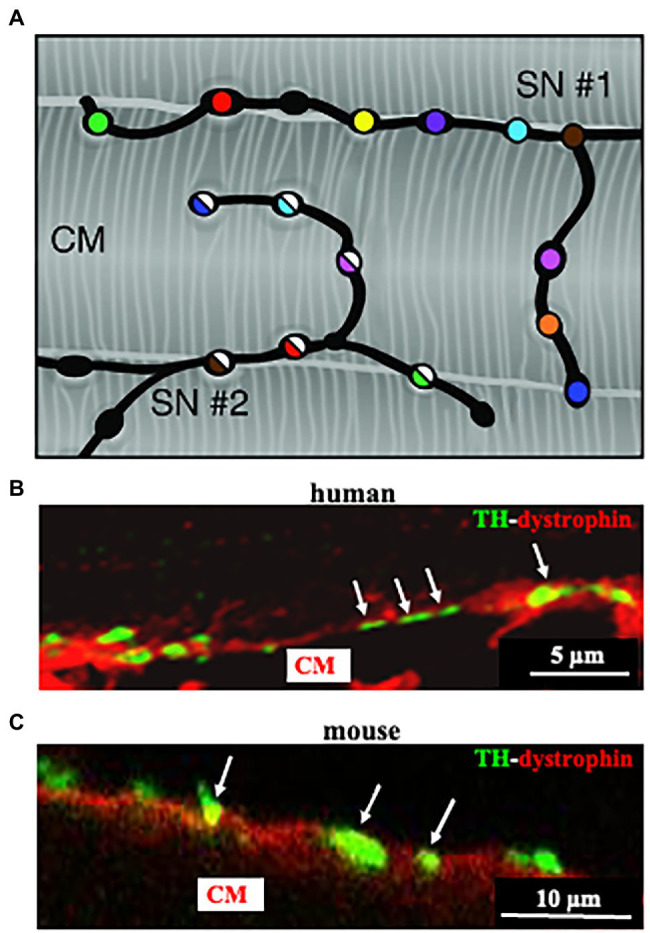
Cardiac sympathetic neurons contact cardiomyocyte membrane at multiples sites. **(A)** Each CM is simultaneously innervated by different neuronal processes (two different processes in the picture are represented by filled or half-filled circles, respectively), all of which are characterized by regularly distributed varicosities, each highlighted by one color. **(B,C)** Confocal immunofluorescence imaging of a thin ventricular section of a normal mouse **(B)**, or human **(C)** heart, stained with antibodies to tyrosine hydroxylase (TH) and dystrophin. Modified with permission from Zaglia and Mongillo (2017) **(A)** Prando et al. (2018) **(B,C)**.

Post-ganglionic SNs descend from sympathetic ganglia, located along the cervical and thoracic spine, and reach the epicardium along the great vessels, before entering the heart wall and distributing throughout the myocardial interstitium (Franzoso et al., 2016). We and others have shown that SN innervation of the mammalian heart is way denser than commonly expected (Franzoso et al., 2016; Zaglia and Mongillo, 2017; Prando et al., 2018; Pianca et al., 2019; Di Bona et al., 2020). Although the topology of heart innervation is species-specific, a common feature, only recently emerged from studies reconstructing the innervation network with high-resolution imaging, is that each CM is enveloped by multiple neuronal processes, which may belong to different branches of the same neuron, but also to different neurons (Zaglia and Mongillo, 2017; Pianca et al., 2019; Figure 3A). Furthermore, given the well-known pearl-necklace morphology of SNs, each process is constituted by regularly distributed enlargements—varicosities—where neurotransmitter vesicles are stored and released from, upon neuronal activation (Figure 3).

The idea that SN varicosities could establish specific contacts with CMs, structurally analogous to the well-known NMJ, dates back several years, but the initial studies failed to detect structured neuro-muscular contacts in the heart, thus endorsing the model whereby SN neurotransmitters “diffuse in wide gaps between nerve processes and cellular targets” (Fawcett and Selby, 1958; Kisch, 1961; Napolitano et al., 1965; Grillo, 1966). In contrast, other studies identified contacts between “unmyelinated SN processes and the CM surface, and the polarization of neurotransmitter vesicles on the side of neuronal varicosities facing the CM” [for a review on the topic see Di Bona et al. (2020)]. These latter studies surmised that neuromuscular transmission occurred at specific interaction sites, postulating that sympathetic neurotransmitters would therefore act on discrete pools of specialized sub-synaptic receptors, well before discovering the molecular identity of ARs (Baxter and Nisbet, 1963; Trautwein and Uchizono, 1963; Thaemert, 1969; Landis, 1976; Klemm et al., 1992; Choate et al., 1993; Di Bona et al., 2020). These conflicting results may be ascribed to the different species analyzed (amphibians vs. mammals), which show a completely different organization of the cardiac innervation network. Furthermore, the earlier studies suffered the lack of methodologies endowed with sufficient resolution and yield to resolve the relations between small, intermittently displaced parts of tortuous neurons and larger myocyte membranes, in thin heart slices.

**Figure 4:**
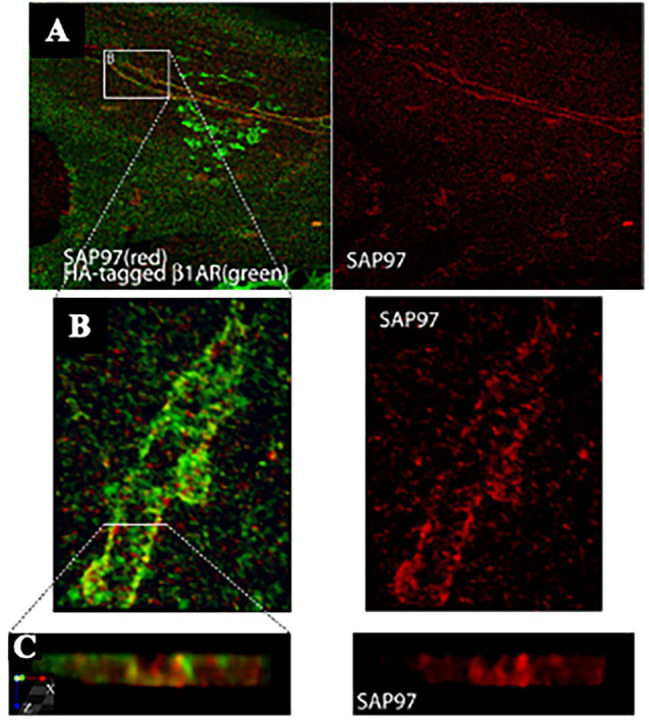
SAP97 and β1-ARs localize to cardiac sympathetic synapses *in vitro*. **(A)** CMs were cocultured with SNs for 7 days, infected with recombinant adenovirus expressing HA-tagged β1-AR, and cultured for an additional 24 h before immunostaining for HA (green) and SAP97 (red). Two-photon image. **(B,C)** Enlargements of the boxed area in **(A)**. Modified with permission from Shcherbakova et al. (2007).

**Figure 5:**
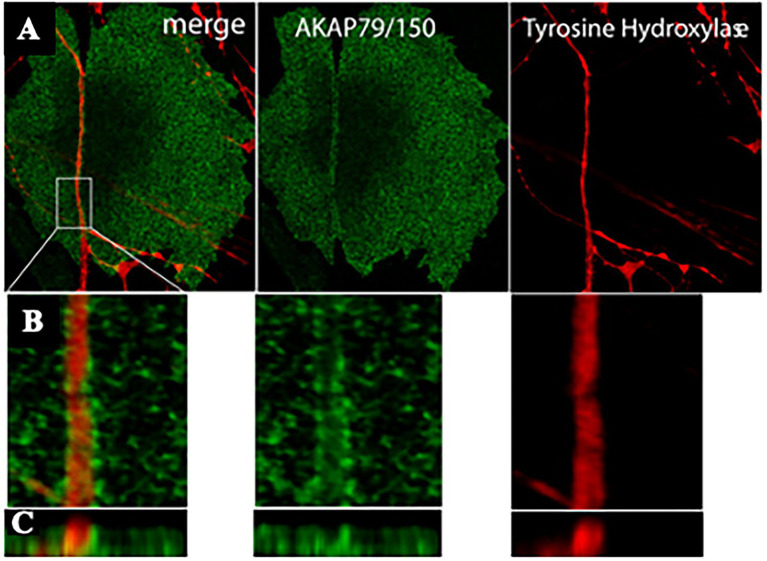
AKAP79/150 accumulates at cardiac sympathetic synapses *in vitro*. **(A)** Colocalization of AKAP79/150 and tyrosine hydroxylase. CMs and SNs were cultured for 7 days and immunostained for tyrosine hydroxylase (red) and AKAP79/150 (green); two-photon image. **(B)** 3D-reconstructed enlarged fragment of the boxed area in **(A)**. **(C)** X-z cross section of the 3D reconstruction. Modified with permission from Shcherbakova et al. (2007).

Reappraisal of cardiac synapses started a decade ago, when Shcherbakova et al. (2007) using co-cultures between SNs and CMs, detected the formation of specialized cell–cell contacts between neuronal varicosities and CM membranes, characterized by specific organization of the post-junctional cell membrane. Remarkably, such sarcolemmal reorganization included membrane invaginations, accumulation of cadherin-catenin complexes [previously shown to have a critical role in stabilization of central synaptic contacts (Bamji et al., 2003), enrichment in β1-ARs and of the scaffold proteins SAP97 and AKAP79/150 (Figures 4, 5; Shcherbakova et al., 2007)]. Some of these aspects were confirmed in intact murine hearts (Shcherbakova et al., 2007). While this evidence supported the existence of a specific junction between SNs and CMs, the differences with the NMJ structural properties were also delineated, including the absence of MuSK, a key player in the organization of the post-synaptic membrane, at the NMJ (Bowen et al., 1998), suggesting therefore that sympathetic synapse formation does not involve agrin-MuSK signaling.

Based on these results, the authors speculated that specific signaling domain on the myocyte plasma membrane, underscored by accumulation of the scaffolding components AKAP79/150 and SAP97, would be required for physiologic signaling of ARs in cardiac tissue. Interestingly, such post-synaptic organization has been described to be depauperated of β2-ARs, which are internalized upon neuronal noradrenaline discharge, thus supporting that “the subtype-specific distribution of β1- and β2-ARs relative to sympathetic synapses” could contribute to signaling specificity (Shcherbakova et al., 2007).

**Figure 6:**
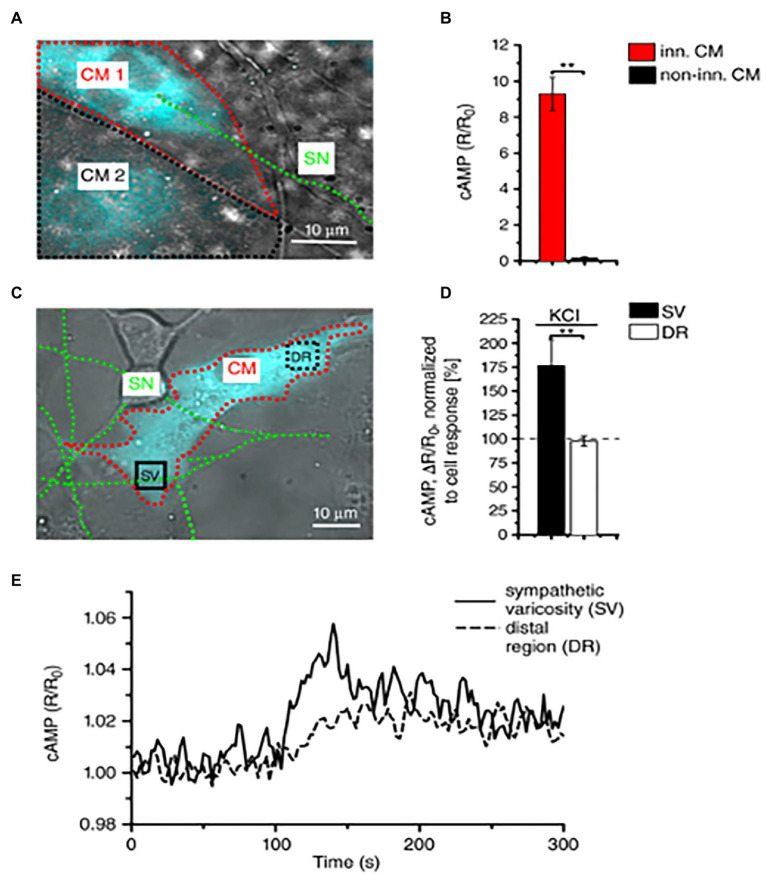
Local activation of cardiomyocytes by innervating neurons. **(A)** Fluorescence image of two adjacent H187-expressing CMs, one of which (CM1, red line) is in direct interaction with a SN (green line), while CM2, highlighted by a black line, is not innervated. **(B)** Statistics of cAMP responses to KCl stimulation of SNs, in innervated vs. non-innervated CMs. Bars indicate SEM (^**^p < 0.01; *n* = 70 CM per group). **(C)** Fluorescence image of a H187-expressing CM innervated by SN processes. cAMP variations were evaluated in the CM delineated by a red line, in regions close to (SV) or far from (DR, distal region) the neurocardiac interaction site. Green line highlights the innervating neuron. **(D)** Statistics of subcellular cAMP variations elicited by neuronal activation in the SV and DR of *n* = 36 CMs with similar neurocardiac arrangements as in **(C)**; bars indicate SEM (^**^p < 0.01). **(E)** Representative trace of cAMP changes calculated in the SV and DR regions of the CM shown in **(C)**. Modified with permission from Prando et al. (2018).

**Figure 7:**
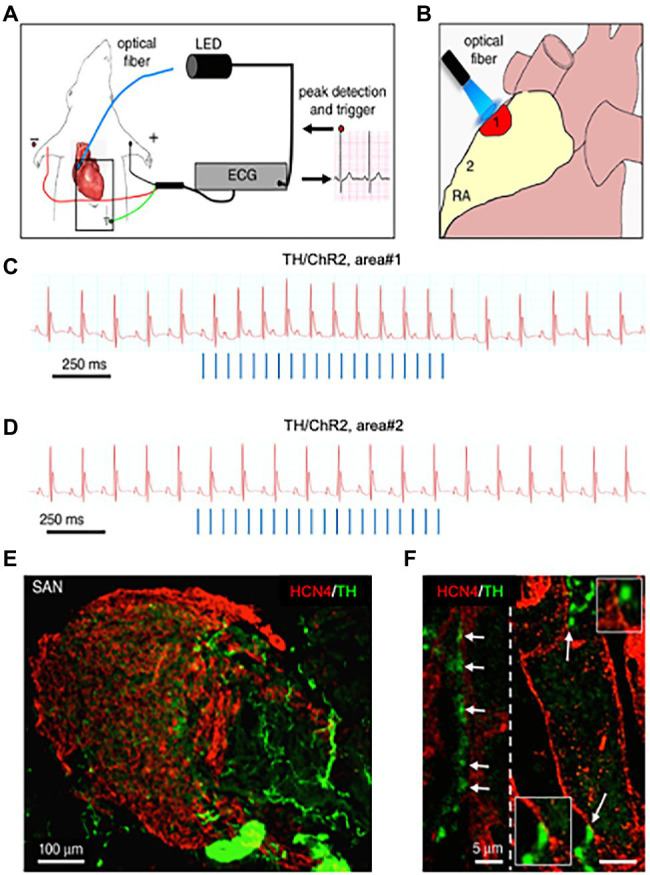
Optogenetic control of cardiac sympathetic neurons *in vivo*. **(A)** Schematic illustration of the neuronal optogenetic set up used for right atrium illumination in open-chest anaesthetized mice. **(B)** Representation of the different photostimulated atrial regions (areas#1–2). **(C)** Representative ECG trace of the optogenetic experiment, showing positive chronotropic response upon photoactivation (blue lines) of the right atrial area#1 in TH/ChannelRhodopsin2 (TH/ChR2) mice. **(D)** Representative trace showing unchanged HR upon illumination of area#2. Behavior of experiments **(C,D)** was observed in *n* = 15 mice. **(E,F)**, area#1 was analyzed with double immunofluorescence with SAN (HCN4) and SN (TH) markers. The magnified image **(F)** highlights the close interaction between SN varicosities and SAN myocytes (left) and an example of multiple neuronal processes interacting with the same myocyte (right). Modified with permission from Prando et al. (2018).

About 10 years later, we combined advanced biotechnologies, including cAMP imaging in “SN-CM” co-cultures, and sympathetic neuron optogenetics *in vivo*, to address neuro-cardiac communication and determine whether the effects of direct synaptic contacts, between neurons and CMs, could be addressed functionally. In line with the previous speculations, by monitoring cAMP, we demonstrated that the tight connection between the neuron and CM membrane enables neuro-cardiac communication to occur *via* specific extracellular signaling microdomains, with several implications on cardiac β-AR activation. First, the tight assembly of the NCJ minimizes extra-synaptic noradrenaline diffusion, rendering the neuronal varicosity a point source of noradrenaline, suited to activate therefore a subset of β-ARs, as shown by the formation of intracellular cAMP gradients, in the CM, originating from the neuronal contact site (Prando et al., 2018). Thus, our data supports the concept whereby spatial cAMP regulation is a downstream effect of the activation of a fraction of β-ARs, located in the limited portion of CM membrane, directly innervated by the neuronal varicosity (Figure 6). Secondly, the low-volume extracellular signaling domain allows (noradrenaline) to activate the receptor at its k_d_ with the number of vesicles released even by a single action potential, as demonstrated by the effect on cAMP elevations in culture. This result was consistent with numerical modeling predictions, previously elaborated using geometrical parameters for the synaptic cleft volume and intermembrane space, in accord with the experimentally determined dimensions (Šćepanović, 2011). Together, these experiments support that the establishment of structured contacts, in which neuro-cardiac communication occurs in a synaptic fashion, is functional for the efficiency of neurogenic regulation of heart function, as predicted by Shcherbakova et al. (2007), and confirmed by the rapid and potent effect of SN photostimulation *in vivo* (Prando et al., 2018; Figure 7).

**Figure 8:**
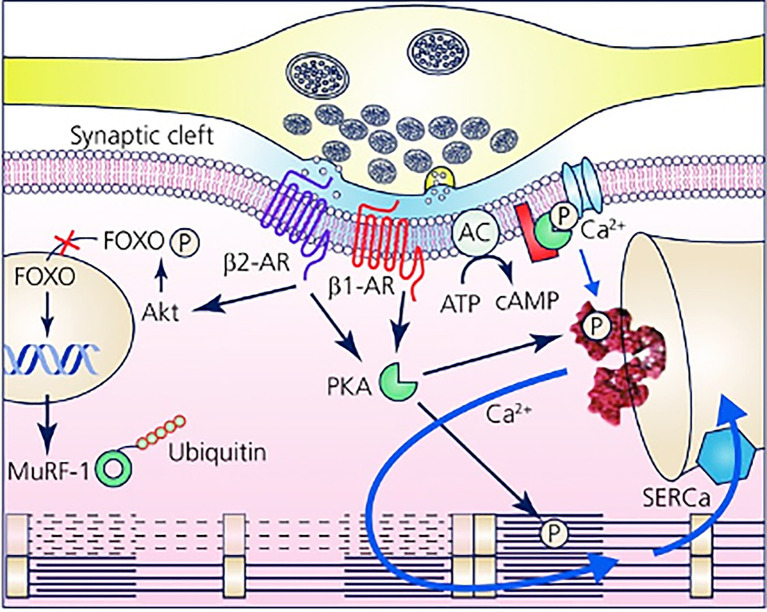
The Neuro-Cardiac Junction is the functional unit of sympathetic neuron-to-cardiomyocytes communication. Schematic of the elements of neuro-cardiac interaction at the varicosity/CM interface. The elements of β-AR dependent signaling activated by noradrenaline released into the synaptic cleft are represented with reference to the β1-AR and β2-AR isotypes. The main downstream targets, respectively, associated with gene regulation of trophic genes (β2-AR) and Ca^2+^ homeostasis (β1-AR) are shown. Modified with permission from Zaglia and Mongillo (2017).

Furthermore, with the notion that NCJs represent the fundamental “signaling units” of neuro-cardiac interaction, summation (integration) of inputs from an increasing number of simultaneously activated NCJs may allow heart regulation through a wide range of tones, spanning from the acute activation, during the stressful “fight-or-flight” reaction, to long-lasting effects, crucial for the maintenance of heart homeostasis (Tao et al., 2011; Zaglia et al., 2013; Zaglia and Mongillo, 2017; Bardsley et al., 2018; Prando et al., 2018; Pianca et al., 2019; Burton et al., 2020; Scalco et al., 2021; Figure 8). In other words, as each CM forms several junctional sites with SNs, and may therefore receive noradrenaline simultaneously from multiple point sources (a varicosity every 1 or 2 mm), it is tempting to speculate that the number of neurons releasing noradrenaline to the same target CM may determine the degree of adrenergic responses across the wide latitude of physiologic regulation (Zaglia and Mongillo, 2017; Figures 3A, 8).

The picture emerging is that, in intact hearts, neuro-cardiac communication involves extra-cellular (first-messenger) signaling microdomains and intracellular (second messenger) micro/nanodomains which combine to control, with exquisite precision and flexibility, CM responses to SN activation. The appreciation that neuro-cardiac communication occurs in defined extra-cellular spaces, that it involves the stimulation of defined receptor pools, each with singular and dynamic distribution on the cell membrane, and that it reflects on a tightly organized series of specific intracellular actions, allows new inference based on experimental data which were, thus far, lacking explanation. This relates to the opening question of the current manuscript: “how can CMs maintain specificity of response to—say—β1- and b2-ARs, even if they are activated from the same extra-cellular agonist and transduced, intracellularly, by the same second messenger?”

To give an example, as discussed above, we have previously demonstrated that the presence of cSN is required to maintain correct CM mass, through constitutive stimulation of β2-AR signaling. However, it has to be taken into account that β1-ARs have higher affinity for noradrenaline than β2-ARs (Devic et al., 2001), and it is thus unlikely that activation of the β2-AR trophic signaling axis is mediated by increased resting levels of noradrenaline in the myocardial interstitium, as this would constitutively activate, and downregulate, the abundant β1-AR isotype. On the other hand, when considering that β2-AR stimulation initiates long-lasting downstream effects (mediated by either cAMP–Epac, PI3K–Akt, or β-arrestin pathways), constitutive control of gene transcription may develop from short repeated neuronal discharges, such as those occurring during normal daily activities (i.e., postural changes, movement; Paula-Gomes et al., 2013; Zaglia and Mongillo, 2017).

Furthermore, the different receptor dynamics may have a role, and in particular, given that β2-ARs, present in correspondence of the NCJ, are internalized upon noradrenaline binding and extruded from the junctional site (Devic et al., 2001; Shcherbakova et al., 2007), we might speculate that the time for reappearance of the receptor, underneath the SN, might represent the limiting factor for neurogenic activation of β2-AR signalling, upon repeated noradrenaline discharges. This mechanism would thus implicate the post-synaptic receptor dynamics in the limitation of receptor activation upon elevated neuronal firing rates, introducing, in other words, a *lowpass* filter in β2-AR dependent signaling (Zaglia and Mongillo, 2017).

This working model may also explain how heart stimulation by SNs is rapidly and simultaneously received by all CMs, with such direct “wired” connection protecting the heart, in physiologic conditions, from heterogeneous adrenergic stimulation which the organ would be exposed to, were the source of NE at variable distance to myocardial targets. The existence of a structured “cardiac synapse”, underscoring the effects of sympathetic stimulation of the heart, has implications in cardiac physiology as well as on the pathophysiology of common cardiovascular diseases, such as heart failure (HF), one of the main causes of death in Europe.

In cardiac physiology, while it is commonly appreciated that catecholamine (norephinephrine, epinephrine) incretion occurs by both sympathetic efferences and the adrenal medulla, the effects of such distinct mechanisms of cardiovascular regulation have not been fully agreed upon. It is tempting to speculate that local neurogenic input, and diffuse stimulation by circulating catecholamines, would activate qualitatively different intracellular signaling compartments, with molecular and functionally different effects. This question is still awaiting answer, which we now have the tools to provide.

In cardiac pathology, it is well understood that HF features alterations in autonomic control of cardiac function, including decreased responsiveness to β-AR agonists, reduced noradrenaline content in sympathetic endings, increased venous spillover of neuronal noradrenaline and its accumulation in the myocardial interstitium (Liang, 2007; de Lucia et al., 2018; Prando, 2018; Ramchandra et al., 2018). Interestingly, most of these features would be explained by structural changes in the NCJ causing the loss of the low-volume intercellular cleft, thus reducing the efficiency of intercellular signaling (Prando et al., 2018), and local neurotransmitter reuptake. In further support of this, our data shows that changes in the NCJ function parallel with post-synaptic membrane disarrangement, SN degeneration and a reduction in the number of neuro-cardiac contacts (Prando et al., 2018). In the context of the complex organization of cAMP signaling in CMs, this scenario would likely impinge on the degree of adrenergic stimulation, and might result in uneven activation of cAMP signaling in different subcellular domains (for reference see Figures 1, 2).

## Is Dystrophin a Structural Component of the Neuro-Cardiac Connection?

Our data surmises that the molecular architecture of the cell–cell interaction site may influence the efficiency of neuro-cardiac communication. At the time being, while the components of the NCJ are still partially unresolved, the observation that in culture, the interaction between SNs and CMs is stable in time, as opposed to that SNs establish with, e.g., cardiac fibroblasts (Pianca et al., 2019), suggests that CM-specific structures may play a role at the intercellular contact site. With reference to the well-described NMJ, we made the hypothesis that dystrophin, which is a central organizer of the post-synaptic skeletal muscle membrane, functional for intercellular communication between MNs and myocytes, may also play a role in the NCJ (Rudolf et al., 2014). Consistently, cardiac fibroblasts, as opposed to CMs, do not express dystrophin (Mezzano et al., 2007). In line with the hypothesis that dystrophin may have a role in building the NCJ and tightening, in a strong bond, neurons and CMs, we observed, in co-cultures, that dystrophin and molecular players of the dystroglycan complex were enriched on the CM membrane, at the interaction site with sympathetic processes (Mongillo et al., 2020). Altogether, this data supports the dystroglycan complex, as a component of the NCJ, might have a role in the functional “SN-CM” communication. Interestingly, it has recently been demonstrated that lack of dystrophin, by affecting the subplasmalemmal cytoskeletal organization, leads to disarray of cAMP compartments, reflecting on the efficiency of β-AR stimulation (Brescia et al., 2020). While this was observed in cultured CMs, together with our observations, these results prompt the idea that absence of dystrophin may lead to profound alteration in the local nature of β-AR activators (neurons) and signaling, thus reflecting on neuro-cardiac fidelity.

**Figure 9:**
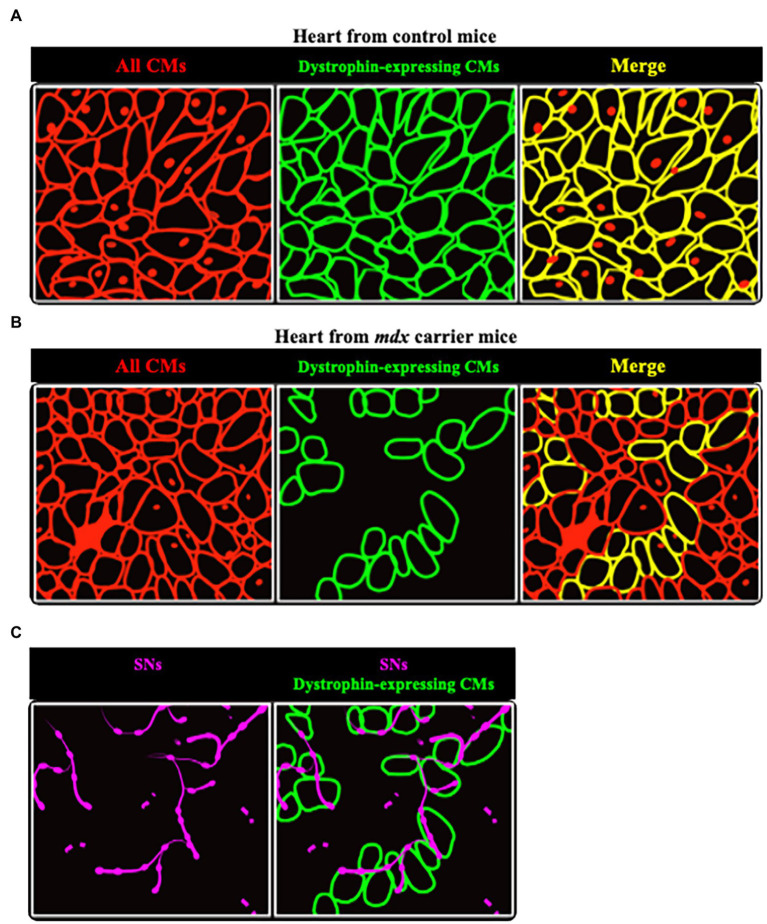
Sympathetic neurons are found selectively in dystrophin-positive areas in hearts from female *mdx* carrier mice. **(A,B)** Representative cartoons showing the comparison between hearts from normal **(A)** and *mdx* carrier **(B)** female mice. While in the former dystrophin is homogeneously expressed by all CMs (identified by red lines), in hearts from *mdx* carriers clusters of dystrophin positive cells (identified by green lines) are intermingled with others lacking dystrophin. **(C)** Areas occupied by dystrophin negative CMs are less innervated, as compared to the positive ones, with neuronal processes (in purple) appear fragmented.

In line with this, hearts of *mdx* mice, a model characterized by the absence of dystrophin expression (Hoffman et al., 1987), had reduced sympathetic innervation density. Most interesting results came from the analysis of hearts from female *mdx* carriers, in which clusters of dystrophin-expressing CMs co-exist with dystrophin-negative ones (Figures 9A,B). In all hearts analyzed, SN processes had lower density in dystrophin-negative regions, compared to the dystrophin-positive ones, identified with immunofluorescence staining (Figure 9C). In addition, the few processes contacting dystrophin-negative CMs had smaller varicosities, compared to those in contact with dystrophin-positive cells (Mongillo et al., 2020).

It is well-appreciated, in several organs innervated by SNs, that the target tissue provides the necessary neurotrophic factors to sustain neuronal survival. In the heart, that CMs could impact on neuronal viability was suggested years ago, although direct demonstration is still lacking. To test whether NCJs, in addition to their role on anterograde (SN-CM) communication, could also impact neurotrophic signaling from CMs to SNs, we set up co-cultures of mixed CMs and SNs from control and *mdx* neonatal mice. In the presence of Nerve Growth Factor in the culture medium, both *mdx* and control neurons extended and branched their processes, developing contacts with CMs, irrespective of the cell genotype. Consistent with the role of CM dystrophin in sustaining “SN-CM” contact, survival of control SNs in co-culture with *mdx* CMs was significantly reduced (Mongillo et al., 2020). Altogether, this data further supports that dystrophin has a key role in bidirectional “SN-CM” communication, and that its ablation reflects on SN trophism and, consequently, on the cardiac innervation pattern.

At this point, one can object that what we observed was in a condition far from that of intact heart, and that previous studies failed to detect accumulation of dystrophin in the portion of the CM membrane innervated by the neuron (as occurring at the NMJ). It has to be noted, however, that while each skeletal muscle fiber establishes a single NMJ, with the MN terminal contacting the cell membrane in a well-defined point, the CM membrane is interspersed by repeating contacts with multiple SN varicosities. This implies that, although dystrophin accumulation may occur in the CM portion contacted by the SN varicosity, differences in dystrophin density along the sarcolemma may be less distinct for the overlap of neighboring postsynaptic membrane portions. Furthermore, we cannot exclude that the dystroglycan complex may contribute to stabilize intercellular contacts independent from selective accumulation at the site of the nervously touched CM membrane.

## Concluding Remarks

The independence of contractions from innervation does not imply that in physiologic conditions the heart beats solo. While neurons, which densely innervate the myocardium, do not ignite contractions, research summarized in this review demonstrates that the finely-built interaction with cardiomyocytes, centered at the NCJ, allows neurons to precisely control multiple cell functions, ranging from contractility, electrophysiology to gene expression. Innervation serves as conductor of the cardiac cellular orchestra, setting the tempo, unifying performers, and shaping the beats of the ensemble. Like a conductor controls interpretation and pace of the music, with gaze and gestures to the performers, neurons do so through direct communication to the cardiomyocytes, guaranteeing the melody and harmony of heartbeats. When diseases break communication between the conductor and the musicians, the orchestra plays out of tune or synchrony, and heartbeats sound turbulent or noisy.

## Author Contributions

MF and LD contributed to manuscript and figure preparation. LV critically discussed data and the review layout. TZ and MM drafted and wrote the manuscript. All authors approved the final version of the manuscript and agree to be accountable for all aspects of the work, in ensuring that questions related to the accuracy or integrity of any part of the work are appropriately investigated and resolved, and that all persons designated as authors qualify for authorship and have been listed.

## Funding

This work was supported by STARS-miniheartwork (UNIPD) to MM and STARS-SKoOP (UNIPD) to TZ.

## Conflict of Interest

The authors declare that the research was conducted in the absence of any commercial or financial relationships that could be construed as a potential conflict of interest.

## Publisher’s Note

All claims expressed in this article are solely those of the authors and do not necessarily represent those of their affiliated organizations, or those of the publisher, the editors and the reviewers. Any product that may be evaluated in this article, or claim that may be made by its manufacturer, is not guaranteed or endorsed by the publisher.

## Abbreviations

β-AR: β-AdrenoReceptor
CM: CardioMyocyte
cSN: cardiac Sympathetic Neuron
GPCR: G-Protein Coupled Receptors
HF: Heart Failure
MN: Motor Neuron
NCJ: Neuro-Cardiac Junction
NMJ: Neuro-Muscular Junction
SN: Sympathetic Neuron
SR: Sarcoplasmic Reticulum
TH: Tyrosine Hydroxylase
T-tubules: Transverse-tubules
